# What are the factors associated with human immunodeficiency virus/sexually transmitted infection screening behaviour among heterosexual men patronising entertainment establishments who engaged in casual or paid sex? – Results from a cross-sectional survey in an Asian urban setting

**DOI:** 10.1186/s12879-016-2088-8

**Published:** 2016-12-19

**Authors:** Raymond Boon Tar Lim, Dede Kam Tyng Tham, Olive N. Y. Cheung, Bee Choo Tai, Roy Chan, Mee Lian Wong

**Affiliations:** 1 Saw Swee Hock School of Public Health, National University of Singapore, Tahir Foundation Building, 12 Science Drive 2, #10-01, Singapore, 117549 Singapore; 2 Department of Sexually Transmitted Infections Control, National Skin Centre, 31 Kelantan Lane, #01-16, Singapore, 200031 Singapore

**Keywords:** Heterosexual men, Entertainment establishment, Human immunodeficiency virus testing, Sexually transmitted infection testing, Risky sexual behaviour, Singapore

## Abstract

**Background:**

Late presentation of human immunodeficiency virus (HIV) is associated with heterosexual transmission, particularly among heterosexual men in Asia. Although data on HIV/sexually transmitted infection (STI) testing behaviour is increasing, information is still lacking among heterosexual men who receive far lesser attention and are generally invisible in HIV/ STI prevention, particularly in the Asian urban setting. The aim of this study was to assess the prevalence of HIV/STI testing among heterosexual men patronising entertainment establishments (EEs) who engaged in casual or paid sex in Singapore, and the factors associated with this behaviour.

**Methods:**

This was a cross-sectional survey involving 604 participants using time location sampling between March and May 2015. For multivariable analysis, we used a mixed effects Poisson regression model with backward stepwise approach to account for clustering by venue and to obtain the adjusted prevalence ratio (aPR) for the association of various factors with HIV/STI testing.

**Results:**

Among 604 at-risk participants, only 163 (27.0%) had gone for HIV or STI testing in the past 6 months. Of this, 83.4% of them specifically underwent HIV testing. In multivariable analysis, HIV/STI testing increased with being non-Chinese (aPR 1.50; 95% CI: 1.08–2.06), having engaged in anal sex with casual or paid partner in the past 6 months (aPR 1.80; 95% CI: 1.27–2.57), number of partners in the past 6 months (aPR 1.03; 95% CI: 1.01–1.05) and HIV knowledge score (aPR 1.11; 95% CI: 1.05–1.16). Among those who reported non-consistent condom use with casual or paid partner, almost half of them (47.9%) perceived that they were at low risk for HIV/STI. Sigmatisation and discrimination was another common barrier for non-testing.

**Conclusions:**

Despite being at risk of HIV/STI, the low prevalence of testing coupled with a high prevalence of risky sexual behaviour among this group of heterosexual men in Singapore calls for a need for HIV/STI prevention interventions in the EE setting. Other than promoting testing and safer sex, the interventions should address the discordance between perceived risk and actual sexual behaviour, in addition to the stigma and discrimination associated with testing for this group.

**Electronic supplementary material:**

The online version of this article (doi:10.1186/s12879-016-2088-8) contains supplementary material, which is available to authorized users.

## Background

Human immunodeficiency virus (HIV)/ Acquired immunodeficiency syndrome (AIDS) was among the top 10 causes of death worldwide in 2012, accounting for 1.5 million deaths [1]. This has been projected to remain similar in 2030 [2]. Majority of the HIV infections in the United States (US) is via homosexual transmission [3] in contrast to Asia where heterosexual transmission predominates [4]. Late presentation of HIV is associated with heterosexual transmission, particularly among heterosexual males in Asia [5]. Late presentation is detrimental to both the infected person and the community’s HIV response. Early and regular testing for HIV and sexually transmitted infections (STIs) is the gateway to treatment, care, and prevention for the at-risk groups because it reduces mortality, morbidity and treatment costs [6]. In developed countries, about 20 to 30% of seropositive individuals were unaware that they were HIV positive [7]. This means that a substantial proportion of people living with HIV get testing and counselling only when they already have advanced clinical disease.

Although data on HIV testing behaviour is increasing from national and sexual behavioural surveys [7], there are still gaps in current evidence. First, most studies focus on HIV testing alone although early testing and timely treatment of STIs can reduce HIV transmission. A meta-analysis demonstrated a 4-fold increased risk of HIV infection with any STI (adjusted odds ratio (aOR) 3.9, 95% CI: 2.8–5.3) [8]. In addition, STIs are common in newly diagnosed HIV patients [9]. Early detection of STIs, particularly those which are curable can potentially decrease HIV transmission. Second, most studies have focused on men who have sex with men (MSM), rather than heterosexual men [10–13]. The available literature provides little data to allow understanding of how sexually active heterosexual men behave in relation to HIV/STI testing [14]. Third, the few studies on sexually active heterosexual men are often from the West [15, 16] and Africa [17–19] where their sexual behaviour and HIV/STI transmission knowledge are likely to be different from that of Asia men [20]. Fourth, to the best of our knowledge, there is no published study that evaluates the factors associated with HIV/STI testing behaviour of heterosexual men patronising entertainment establishments (EEs) who engage in casual or paid sex. EEs are karaoke lounges, bars, pubs, nightclubs and discotheques that provide entertainment activities such as singing, dancing and social drinking [21]. In recent years, sex work has increasingly shifted from brothels to EEs, particularly in Asia [22]. Heterosexual men patronising EEs who engage in casual or paid sex is an important and yet often neglected at-risk group because these men could potentially act as a bridging population for HIV/STI transmission through unprotected sex to their spouses and regular partners, resulting in extensive transmission to the community [23, 24].

Singapore is a multi-ethnic Asia country representing East (Chinese), South-East (Malay) and South Asian (Indian) ethnicities [25]. The percentage of heterosexual Singapore male residents engaging in sexual risk behaviour (defined as having commercial and/or casual sexual exposures) has increased from 4.7% in 1989 [26] to 18.5% in 2007 [27]. One common setting where casual and paid sex takes place in Singapore is in the EEs [23, 24]. Similar to previous years, HIV cases were predominantly males in Singapore, with a male to female ratio of 12:1 in 2014 [28]. Heterosexual transmission accounted for 46.3% of all cases in 2014, while homosexual and bisexual transmission accounted for 48.3% [28]. Late diagnosis of HIV remains a major public health issue here. In 2014, 49% of the new cases already had late-stage HIV infection when they were diagnosed, compared to 41% in 2013 and 48% in 2012 [28]. Of the new cases in 2014, only 14% were detected through voluntary testing, and this was lowest for heterosexual men among the priority groups [28]. Apart from the different modes of sexual transmission, there were ethnic differences in HIV screening behaviour among the general population in Singapore, with non-Chinese (consisting of mainly Malays and Indians) being more likely than the Chinese to undergo screening [29, 30]. Till now, it is unclear whether these ethnic differences also existed among sexually active heterosexual men. The aim of this paper was to assess the prevalence of HIV/STI testing behaviour among heterosexual men patronising EEs who engaged in casual or paid sex in Singapore, and the factors associated with this behaviour. The findings would help provide information for policy makers and public health professionals to enhance their understanding on HIV/STI testing behaviour for future planning of HIV/STI prevention interventions for this group of heterosexual men.

## Methods

### Study design and population

This was a cross-sectional survey between March and May 2015 involving heterosexual men patronising EEs who engaged in casual or paid sex. It was part of a study to assess the efficacy of a health promotion and STI/HIV prevention programme in EEs. Study participants had to be either a Singapore citizen or permanent resident between the ages of 21 to 69 years old who had (i) engaged in vagina, oral or anal sex with either a casual or paid female partner in the past 6 months and (ii) patronised the EEs in the study sites at least once in the past 6 months. The casual or paid female partner need not come from the EEs in the study sites that the heterosexual man has patronised. Homosexual (defined as ever having intercourse with a partner of the same gender) and bisexual (defined as ever having intercourse with partners of both gender) men were excluded from the study.

### Sample size and sampling method

Participants were sampled in equal proportions from two well defined geographical sites in Singapore (Clarke Quay and Tanjong Pagar) which had similar estimated population size of heterosexual men who patronised EEs using the enumeration method recommended by the World Health Organisation [31]. To detect a difference of 10% in the proportion of HIV/STI testing from the prevalence of 30% in 2008 [32] to 40% for this study using one sample, the estimated minimum sample size required was 300 to give a power of 80%, a level of significance (alpha) of 0.05 (two sided), a cluster design with eight clusters and an intra-class correlation of 0.01. Assuming a participation rate of 85% based on previous local study [32] and that about 60% of the heterosexual men patronising EEs engaged in casual or paid sex [32], a minimum of 590 men were required. These men were selected using time location sampling at different times of the day at different EEs during the venues’ operating hours from 4 pm to 12 midnight on weekdays and weekends over a 3-month period to reduce selection bias. Three hundred and two men from 8 EEs in Clarke Quay and the same number from 20 EEs in Tanjong Pagar were selected. Field recruiters waited outside specific EEs according to a pre-determined time location sampling frame and recruited the men who were about to step into or out from these places. To reduce social desirability bias, we acknowledged their difficulties in practising safer sex and stressed the importance of responding honestly because their responses would be used for programme improvement. We also used frequency-based questions rather than leading question in the questionnaires. For example, instead of asking “Do you use condom consistently?” (options of ‘no’ and ‘yes’), we asked “how often do you use condoms?” (options of ‘not applicable’, ‘never’, ‘sometimes’ and ‘always’). The recruitment flow process is available in Additional file 1: Appendix I.

### Data collection and ethics approval

The study consisted of a self-administered anonymised questionnaire in English approved by the National University of Singapore Institutional Review Board (approval certificate number NUS 2159). The outcome was HIV or STI testing behaviour for the past 6 months. Participants were asked specifically whether it was HIV testing alone, STI testing (excluding HIV) or both HIV and STI testing. Participants were subsequently dichotomised into two groups, those who had gone for either HIV or STI testing in the past 6 months versus those who did not. Factors assessed included sociodemographics, sexual behaviour and HIV knowledge. For sociodemographic factors, this included age (years), ethnicity (Chinese versus non-Chinese), marital status, highest education level, housing type and occupation. For sexual behaviour, this included number of partners (both regular and casual/paid) in the past 6 months, whether engaged in anal sex with casual or paid partner in the past 6 months defined as the penis entering the female partner’s anus, type of casual/paid partners in the past 6 months (casual partner referred to a woman whom the man had a one-night stand with and not involving any commercial transaction; female entertainment worker referred to a woman from the EEs, e.g. beer promoters, karaoke singers, dancers, massage workers or hostesses; and sex worker from brothels referred to a woman involved in selling sex from a brothel), type of regular partners in the past 6 months (referred to wife or girlfriend/mistress), partner asking to use a condom all the time in the past 6 months, consistent condom use with casual or paid partner in the past 6 months and condom use at last sex with a casual or paid partner. For HIV knowledge, this was determined by the HIV Knowledge Questionnaire (HIV-KQ-18) on HIV transmission, prevention and misconceptions about HIV infection [33]. It consisted of 18 items categorised into “true”, “false” and “don’t know”. A score of 1 was assigned to each correct answer, and no score was given for ‘don’t know’ and incorrect answer. Scores were summed (range: 0 to 18), with higher scores indicating greater knowledge [33]. This instrument was reported to be reliable [33], and the Cronbach’s alpha was 0.84 for this study, indicating high internal consistency.

### Statistical analyses

All questionnaires were included in the analysis as they contained completed information on the outcome, i.e. HIV/STI testing behaviour. We described the sociodemographic factors, HIV knowledge and sexual behaviour factors by ethnicity and HIV/STI testing behaviour. Categorical variables were compared with the use of the chi-square test, ordinal variables with the Mann-Whitney U-test and continuous variables with the independent-sample t test. *P* value of ≤0.05 was taken as statistically significant. We obtained the crude prevalence ratio (PR) and 95% confidence interval (CI) for the association of these factors with HIV/STI testing behaviour. A mixed effects Poisson regression model was used to account for clustering by venue. To identify the independent factors, those with p value ≤0.10 from the bivariate analysis were selected for multivariable analysis. Backward stepwise approach was performed to obtain the adjusted PR (aPR) and 95% CI, where only all variables included in the final model have *p* ≤ 0.05. We also compared the reasons for not going for HIV/STI testing between the Chinese and the non-Chinese as well as between those who reported consistent condom use and those who did not. All statistical analyses were performed using STATA version 11.2 (Stata Corp, College Station, TX).

## Results

**Fig. 1:**
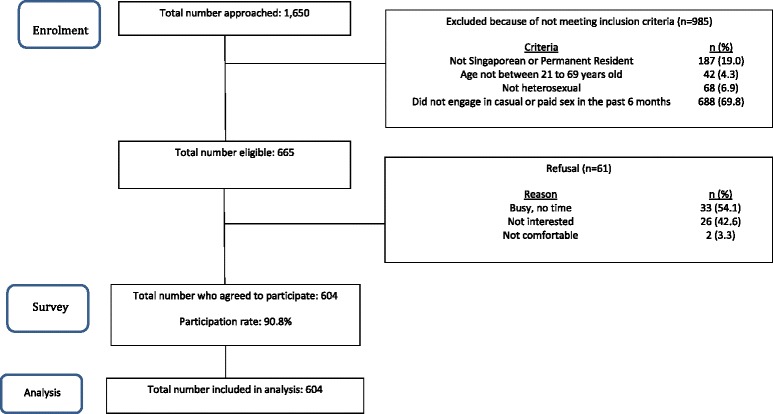
Study flow chart for heterosexual men patronising entertainment establishments who engaged in casual or paid sex

A total of 604 heterosexual men patronising EEs who engaged in casual or paid sex was recruited, giving a participation rate of 90.8% (Fig. 1). There was no significant difference in the distribution of venue (*p* = 0.86) and ethnicity (*p* = 0.90) among those who agreed to participate and those who refused. On comparing these heterosexual men from the 2 geographical sites, there was no statistical difference in the outcome, i.e. HIV/STI testing behaviour (*p* = 0.31) (Additional file 2: Appendix II). Out of 604 men, 163 (27.0%) had gone for HIV or STI testing in the past 6 months. Of the 163, 136 (83.4%) of them specifically underwent HIV testing. Among the 163 who underwent HIV/STI testing, 17 (10.4%) reported STI-related symptoms or signs i.e. discharge from penis, ulcer or sore on penis, growth on penis and pain on passing urine. Of the 17, 12 of them (70.6%) sought medical consultation and were given treatment. The top reason given for not seeking medical consultation was the perception that the symptom or sign was not serious.

Table 1 shows the distribution of sociodemographic factors, HIV knowledge and sexual behaviour by ethnicity. The Chinese had a higher median HIV knowledge score (14; IQR 11–15) than the non-Chinese (13; IQR 9–15). There were a greater proportion of risky sexual behaviours in the non-Chinese compared to the Chinese. The non-Chinese reported a higher median number of partners in the past 6 months (5; IQR 3–6) compared to the Chinese (4; IQR 2–6), a higher proportion had engaged in anal sex with casual or paid partner in the past 6 months (22.5%) compared to the Chinese (14.1%) and a lower proportion (51.1%) reported condom use at last sex with a casual or paid partner compared to the Chinese (65.8%).

**Table 1:** Comparison of sociodemographics, HIV knowledge and sexual behaviour by ethnicity

Factor	Total (*n* = 604)	Chinese (*n* = 377)	Non-Chinese (*n* = 227)	*P* value
Sociodemographics				
Marital status				
Single	492 (81.5)	309 (82.0)	183 (80.6)	0.68
Married	112 (18.5)	68 (18.0)	44 (19.4)	
Highest education level^a^				
No formal education/primary/secondary	90 (15.0)	49 (13.0)	41 (18.2)	<0.001
Institute of technical education/A Level/ Diploma	332 (55.1)	194 (51.5)	138 (61.3)	
University/Post-graduate	180 (29.9)	134 (35.5)	46 (20.5)	
Housing type				
1–3 room public housing	91 (15.1)	51 (13.5)	40 (17.6)	0.24
4–5 room public housing	345 (57.1)	214 (56.8)	131 (57.7)	
Private property	168 (27.8)	112 (29.7)	56 (24.7)	
Occupation				
Not currently employed	102 (16.9)	62 (16.4)	40 (17.6)	0.22
Blue-collar job	157 (26.0)	90 (23.9)	67 (29.5)	
White-collar job	345 (57.1)	225 (59.7)	120 (52.9)	
Age in years, median (IQR)	26 (23–32)	26 (24–32)	26 (23–32)	0.48
HIV knowledge				
HIV-KQ-18 Knowledge Score, median (IQR)	14 (10–15)	14 (11–15)	13 (9–15)	0.01
Sexual behaviour				
Number of partners in the past 6 months, median (IQR)	4 (2–6)	4 (2–6)	5 (3–6)	0.003
Engaged in anal sex with casual or paid partner in the past 6 months				
No	500 (82.8)	324 (85.9)	176 (77.5)	0.008
Yes	104 (17.2)	53 (14.1)	51 (22.5)	
Type of casual/paid partners in the past 6 months				
More than 1 type of casual/paid partner	156 (25.8)	104 (27.6)	52 (22.9)	0.63
Sex workers from brothels only	27 (4.5)	16 (4.2)	11 (4.8)	
Casual partners only	370 (61.3)	225 (59.7)	145 (63.9)	
Female entertainment workers only	51 (8.4)	32 (8.5)	19 (8.4)	
Type of regular partners in the past 6 months				
Without any regular partner	330 (54.6)	204 (54.1)	126 (55.5)	0.88
With wife as regular partner only	53 (8.8)	32 (8.5)	21 (9.2)	
With girlfriend/mistress as regular partner only	198 (32.8)	125 (33.2)	73 (32.2)	
With both wife and girlfriend/mistress as regular partners	23 (3.8)	16 (4.2)	7 (3.1)	
Partner asking to use a condom all the time in the past 6 months				
No	286 (47.4)	180 (47.8)	106 (46.7)	0.80
Yes	318 (52.6)	197 (52.2)	121 (53.3)	
Consistent condom use with casual or paid partner in the past 6 months				
No	320 (53.0)	194 (51.5)	126 (55.5)	0.33
Yes	284 (47.0)	183 (48.5)	101 (44.5)	
Condom use at last sex with a casual or paid partner				
No	240 (39.7)	129 (34.2)	111 (48.9)	<0.001
Yes	364 (60.3)	248 (65.8)	116 (51.1)	

All figures in the table refer to frequency (column percentage) unless otherwise indicated

^a^Contains missing value of 2 for highest education level

Table 2 shows the proportion and crude PRs of heterosexual men who underwent HIV/STI testing in the past 6 months versus those who did not by the various factors. Compared with the Chinese, the non-Chinese had a higher prevalence of having gone for testing (PR 1.49; 95% CI: 1.09–2.02). Compared with those who were not currently employed, those holding a white-collar job had a higher prevalence of having gone for testing (PR 1.77; 95% CI: 1.06–2.96). Compared with those who did not engage in anal sex with casual or paid partner in the past 6 months, those who had done so had a higher prevalence of having gone for testing (PR 1.89; 95% CI: 1.34–2.66). HIV/STI testing behaviour also increased with HIV knowledge score (PR 1.10; 95% CI: 1.04–1.15) and number of partners in the past 6 months (PR 1.04; 95% CI: 1.02–1.06).

**Table 2:** Proportion and crude prevalence ratio of heterosexual men who underwent HIV/STI testing in the past 6 months versus those who did not by various factors

Factor	Gone for HIV/STI testing		Crude PR (95% CI)	*P* value
	Yes (*n* = 163)	No (*n* = 441)		
Sociodemographics				
Ethnicity				
Chinese	86 (22.8)	291 (77.2)	Referent	
Non-Chinese	77 (33.9)	150 (66.1)	1.49 (1.09–2.02)	0.01
Marital status				
Single	129 (26.2)	363 (73.8)	Referent	
Married	34 (30.4)	78 (69.6)	1.16 (0.79–1.69)	0.45
Highest education level^a^				
No formal education/primary/secondary	25 (27.8)	65 (72.2)	Referent	
Institute of technical education/A Level/ Diploma	83 (25.0)	249 (75.0)	0.90 (0.58–1.41)	0.64
University/Post-graduate	54 (30.0)	126 (70.0)	1.08 (0.67–1.74)	0.75
Housing type				
1–3 room public housing	22 (24.2)	69 (75.8)	Referent	
4–5 room public housing	91 (26.4)	254 (73.6)	1.09 (0.68–1.74)	0.71
Private property	50 (29.8)	118 (70.2)	1.23 (0.75–2.03)	0.42
Occupation				
Not currently employed	17 (16.7)	85 (83.3)	Referent	
Blue-collar job	44 (28.0)	113 (72.0)	1.68 (0.96–2.94)	0.07
White-collar job	102 (29.6)	243 (70.4)	1.77 (1.06–2.96)	0.03
Age in years, median (IQR)	27 (24–32)	26 (23–30)	1.02 (1.00–1.03)	0.11
HIV knowledge				
HIV-KQ-18 Knowledge Score, median (IQR)	15 (12–16)	13 (10–15)	1.10 (1.04–1.15)	0.001
Sexual behaviour				
Number of partners in the past 6 months, median (IQR)	5 (3–6)	4 (2–5)	1.04 (1.02–1.06)	<0.001
Engaged in anal sex with casual or paid partner in the past 6 months				
No	117 (23.4)	383 (76.6)	Referent	
Yes	46 (44.2)	58 (55.8)	1.89 (1.34–2.66)	<0.001
Type of casual/paid partners in the past 6 months				
More than 1 type of casual/paid partner	44 (28.2)	112 (71.8)	Referent	
Sex workers from brothels only	7 (25.9)	20 (74.1)	0.92 (0.41–2.04)	0.84
Casual partners only	103 (27.8)	267 (72.2)	0.99 (0.69–1.40)	0.94
Female entertainment workers only	9 (17.6)	42 (82.4)	0.63 (0.31–1.28)	0.20
Type of regular partners in the past 6 months				
Without any regular partner	79 (23.9)	251 (76.1)	Referent	
With wife as regular partner only	17 (32.1)	36 (67.9)	1.34 (0.79–2.26)	0.27
With girlfriend/mistress as regular partner only	58 (29.3)	140 (70.7)	1.22 (0.87–1.72)	0.24
With both wife and girlfriend/mistress as regular partners	9 (39.1)	14 (60.9)	1.63 (0.82–3.26)	0.16
Partner asking to use a condom all the time in the past 6 months				
No	80 (28.0)	206 (72.0)	Referent	
Yes	83 (26.1)	235 (73.9)	0.93 (0.69–1.27)	0.66
Consistent condom use with casual or paid partner in the past 6 months				
No	80 (25.0)	240 (75.0)	Referent	
Yes	83 (29.2)	201 (70.8)	1.17 (0.86–1.59)	0.32
Condom use at last sex with a casual or paid partner				
No	65 (27.1)	175 (72.9)	Referent	
Yes	98 (26.9)	266 (73.1)	0.99 (0.73–1.36)	0.97

All figures in the table refer to frequency (row percentage) unless otherwise indicated

^a^Contains missing value of 2 for highest education level

Table 3 shows the multivariable associations of the various factors with HIV/STI testing behaviour. There was no notable statistical interaction among the factors. HIV/STI testing increased with being non-Chinese (aPR 1.50; 95% CI: 1.08–2.06), having engaged in anal sex with casual or paid partner in the past 6 months (aPR 1.80; 95% CI: 1.27–2.57), number of partners in the past 6 months (aPR 1.03; 95% CI: 1.01–1.05) and HIV knowledge score (aPR 1.11; 95% CI: 1.05–1.16). Occupation was not significant in the final model.

**Table 3:** Multivariable associations between the various factors and HIV/STI testing amongst heterosexual men in the past 6 months

Factor	Adjusted PR (95% CI)
Ethnicity	
Chinese	Referent
Non-Chinese	1.50 (1.08–2.06)
Engaged in anal sex with casual or paid partner in the past 6 months	
No	Referent
Yes	1.80 (1.27–2.57)
Number of partners in the past 6 months	1.03 (1.01–1.05)
HIV-KQ-18 Knowledge Score	1.11 (1.05–1.16)
Occupation	
Not currently employed	Referent
Blue-collar job	1.43 (0.81–2.52)
White-collar job	1.47 (0.87–2.46)

**Fig. 2:**
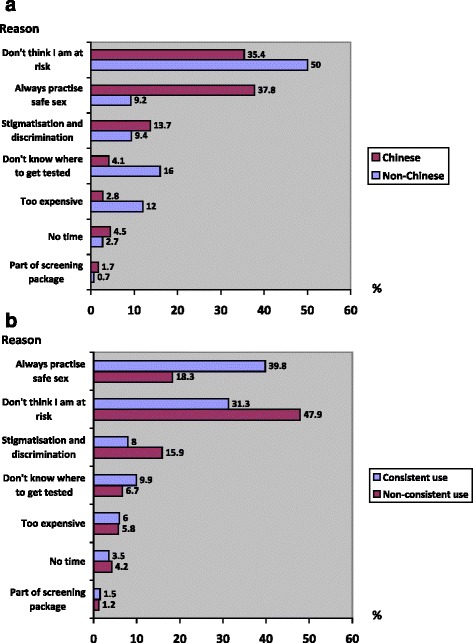
**a** Reasons of heterosexual men not going for HIV/STI testing by ethnicity (*n* = 441). **b** Reasons of heterosexual men not going for HIV/STI testing by consistent condom use with casual or paid partner in the past 6 months (*n* = 441)

The top three reasons for not going for HIV/STI testing among these heterosexual men were “don’t think I am at risk” (40.4%), “always practise safe sex” (28.1%) and “stigmatisation and discrimination” (12.3%). Fig. 2a show the reasons of these heterosexual men not going for testing by ethnicity. Among the Chinese, the top 3 reasons were “always practise safe sex” (37.8%), “don’t think I am at risk” (35.4%), “stigmatisation and discrimination” (13.7%). As for the non-Chinese, the top 3 reasons were “don’t think I am at risk” (50.0%), “don’t know where to get tested” (16.0%) and “test too expensive” (12.0%). Fig. 2b shows the reasons of these heterosexual men not going for testing by consistent condom use with casual or paid partner in the past 6 months. Among those who reported consistent use, the two most common reasons were “always practise safe sex” (39.8%) followed by “don’t think I am at risk” (31.3%). As for those who reported non-consistent use, this was “don’t think I am at risk” (47.9%) followed by “always practise safe sex” (18.3%).

## Discussion

Our study showed that 27.0% of the participants had gone for HIV/STI testing in the past 6 months. This prevalence was lower compared to MSM from gay venues and hotspots in Cambodia (65.1%) [10], MSM living in Bangkok or Chiang Mai, Thailand (74.1%) [11], MSM from bar, bathhouse, park, and internet setting in Beijing, China (56.2%) [12] and MSM from bar, brothel and internet setting in Chongqing, China (58.0) [13] but higher than heterosexual men who attended the STI clinics in China (9.3%) [20]. One reason for this phenomenon is probably due to the social movements and advocacy from various interest groups [14] that has led to more research and corresponding delivery of interventions for the MSM [34]. This is in contrast to heterosexual men, who have received far lesser attention and are generally invisible in HIV/STI prevention [35]. The low prevalence of HIV/STI testing coupled with a high prevalence of risky sexual behaviour among heterosexual men patronising EEs who engaged in casual or paid sex calls for a need for HIV/STI prevention programmes in the EE setting. Other than promoting safer sex, one key component of the programme should include promoting HIV/STI testing behaviour and making testing more accessible in the EE setting.

HIV/STI testing increased with number of partners in our study. This is similar to other studies where HIV testing increased with higher number of past year sex partners among male motorbike taxi drivers (92.1% were heterosexual) in urban Vietnam [36] and among the general heterosexual male population in Italy [16]. For having engaged in anal sex, our result showed an almost two-fold significant association with HIV/STI testing although this was not observed in heterosexual men who attended the STI clinics in China [20]. We did not find an association of consistent condom use with HIV/STI testing in our study. Other studies have reported mixed findings with no association found among the general heterosexual male population in Italy [16] but with an association in heterosexual men who attended the STI clinics in China [20].

The observed association between HIV/STI testing and HIV knowledge score in our study was consistent with two other cross-sectional studies, namely Scott-Sheldon LA et al., [18] and Haile BJ et al, 2007 [19]. The cross-sectional study design prevents strong inferences regarding the directionality of this association. Although knowledge could heighten an individual’s perceived risk towards HIV/STI infection, hence affecting his decision to undergo testing; having undergone testing could also mean that the individual, having been counselled, would lead to increased HIV knowledge.

HIV/STI testing behaviour was more common among the non-Chinese in our study. Other studies in Singapore have also reported that the non-Chinese were more likely to take up HIV screening than the Chinese [29, 30]. In 2008, Singapore implemented HIV voluntary opt-out screening (VOS) for hospitalised adults. The Chinese were more likely to opt-out compared to the non-Chinese in the second largest acute care general hospital in Singapore in 2009–2010 [29]. In another major tertiary hospital in Singapore, 94.9% of the Chinese opt-out of the HIV VOS, compared to 84.1% of Indians and 86.7% of Malays [30]. The reasons for the ethnic differences in Singapore remain unclear. Although housing type and occupation were not significant determinants of testing behaviour in bivariate and multivariable analysis respectively, we could not exclude the presence of other unmeasured confounders related to socioeconomic status. In addition, ethnicity could be a proxy of other sociocultural variables not measured in our study such as social network and cultural norms. Further studies are needed to determine the reasons for these ethnic differences in testing behaviour as to whether it is a proxy of other variables or whether such variations do indeed exist.

Our study identified two specific areas of need for HIV/STI intervention targeting heterosexual men patronising EEs who engaged in casual or paid sex in Singapore. First, there was a clear discordance between perceived risk and actual sexual behaviour in this group. Our results have showed that among those who reported non-consistent condom use, almost half of them perceived themselves to be at low risk for HIV/STI and another one-fifth perceived that they practised safe sex. These were the two most common reasons given for non-testing despite them engaging in risky unprotected sexual behaviour. Low risk perception is an important barrier to testing as people who do not perceive themselves to be at risk of infection are less likely to go for testing and also take preventive measures such as condom usage [6]. Such discordance has been well reported in other HIV at-risk groups such as sexually active adolescents, MSM and female sex workers in Asia [37–39]. For instance, adolescents who engaged in unprotected sex and yet held low risk perception were less likely to go for screening due to their lack of perceived susceptibility to HIV/STI compared to those who had higher risk perception [38]. Although this discordance has also been reported among sexually active heterosexual men in US [40], evidence is lacking for heterosexual men patronising EEs who engaged in casual or paid sex. Our study adds to this limited pool of evidence, indicating a need to address the discordance for this at-risk group of heterosexual men who is often invisible in HIV/STI prevention.

Second, it is also important to address the stigmatisation and discrimination associated with testing. This was the third most common reason for non-testing behaviour among these heterosexual men, including the Chinese. Both the concern of being embarrassed when going for testing and the fear of stigma, discrimination, rejection from a positive diagnosis have been highlighted as more important than fear of death or illness in the West [6]. Although stigma is often considered a major barrier to effective responses to HIV/STI prevention programme, stigma reduction efforts are often relegated to the bottom of programme priorities [41]. In-depth interviews with the heterosexual men in our study have revealed similar concerns such as being judged by family and peers; fear of backlash and unacceptance if they were to go for screening and were tested positive. Therefore it would be important to shift the focus of promotion of HIV/STI screening from a disease-centric to a positive sexual well-being approach for this group.

Most interventions studies to reduce stigma and discrimination on HIV testing are targeted at MSM, sex workers and injection drug users [41–43]. Interventions to improve HIV/STI testing are generally lacking among heterosexual men, particularly for this group who patronises EEs and engages in casual or paid sex [6, 14]. Future interventions for them should use strategies to enhance their risk perception and encourage HIV testing as well as to address stigma and discrimination associated with testing. These interventions should be held in EEs so as to reach out to the captive audience of the at-risk group of heterosexual men frequenting these establishments and their casual partners, as well as to female entertainment workers and other employees of the EEs. Appropriate educational activities to appeal to the heterosexual men could take the form of education entertainment (‘edutainment’) where health messages on sexual well-being, HIV testing and safer sex are incorporated into entertainment activities such as short talk shows and stage performances. This approach is likely to gain acceptance among the heterosexual men, the EE management and owners because it is holistic, non-disease centric, non-stigmatising and non-judgemental. Edutainment such as fashion photography and online YouTube videos of male celebrities and gay personalities promoting testing has been found to be effective in reducing stigma and increasing HIV screening among MSM in Thailand [44]. Strategies to enhance HIV risk perception should include a personalised risk assessment tool delivered through a password-protected interactive web portal to help the heterosexual men to assess their HIV risk. An intervention programme targeted at the African-American general heterosexual male population in the US have found the use of personalised risk assessments to be effective in increasing risk perception and HIV screening uptake rates [45].

### Limitations and strengths

This study has some limitations, one of which is the social desirability bias on sexual behaviour among participants. We assured participants that their responses were anonymous and all fieldwork recruiters have been trained to follow the steps highlighted in the methodology section to reduce social desirability bias. Second, this study is limited by its cross-sectional design which cannot infer causal relationships. Third, the use of HIV-KQ-18 cannot be considered a comprehensive measure of HIV-related knowledge since it assesses mainly the various transmission modes of HIV and that the questionnaire has not been validated in the Singapore heterosexual male population.

This study fills the current gaps in knowledge pertaining to HIV/STI testing behaviour among heterosexual men patronising EEs who engage in casual or paid sex, an increasingly important group for HIV/STI transmission in Asia. Careful sampling procedures were applied to ensure a representative random sample so that the findings could be generalised to this group in Singapore. The high response rate and use of time location sampling also supports the generalisability of the study findings to the EEs in the study sites.

## Conclusions

In conclusion, the low prevalence of HIV/STI testing coupled with a high prevalence of risky sexual behaviour among heterosexual men patronising EEs who engaged in casual or paid sex in our study indicates a need for HIV/STI prevention programme in the EE setting in Singapore. Other than promoting testing and safe sex, there are two specific areas of need to address for this group, namely the discordance between perceived risk and actual sexual behaviour as well as the stigma and discrimination associated with HIV/STI testing.

## Additional files

Additional file 1: Appendix I. Recruitment process for heterosexual men patronising entertainment establishments (EEs) who engaged in casual or paid sex. (DOC 94 kb)
Additional file 2: Appendix II. Outcome, sociodemographic factors, HIV knowledge and sexual behaviour amongst heterosexual men by geographical site. (DOC 114 kb)

## Abbreviations

AIDS: Acquired immunodeficiency syndrome
aOR: Adjusted odds ratio
aPR: Adjusted prevalence ratio
CI: Confidence interval
EEs: Entertainment establishments
HIV: Human immunodeficiency virus
HIV-KQ-18: HIV Knowledge Questionnaire
MSM: Men who have sex with men
OR: Odds ratio
PR: Prevalence ratio
STIs: Sexually transmitted infections
US: United States
VOS: Voluntary opt-out screening
