# Supplementary material for: What are the factors associated with human immunodeficiency virus/sexually transmitted infection screening behaviour among heterosexual men patronising entertainment establishments who engaged in casual or paid sex? – Results from a cross-sectional survey in an Asian urban setting
Source: BMC Infect Dis. 2016 Dec 19;16:763. doi: 10.1186/s12879-016-2088-8 (PMC5168707; doi:10.1186/s12879-016-2088-8)
Supplement: Additional file 1: — Appendix I. Recruitment process for heterosexual men patronising entertainment establishments (EEs) who engaged in casual or paid sex. (DOC 94 kb) [file 12879_2016_2088_MOESM1_ESM.doc]

# Additional file 1: Appendix I. Recruitment process for heterosexual men patronising entertainment establishments (EEs) who engaged in casual or paid sex

Fieldwork recruiters wait outside the specific EE according to the pre-determined time location sampling frame

Approach man who is about to step into or out from the EE

Initiate informal conversation with the man

Elicit non-sensitive information that is part of the inclusion criteria such as nationality and age

Fulfil the non-sensitive inclusion criteria

Does not fulfil the non-sensitive inclusion criteria

Thank the man for his time and approach another man

Pass the man a short screening questionnaire containing sensitive questions on sexual orientation, types of sexual acts and types of sexual partner for the past 6 months

The man is left alone to answer the short screening questionnaire which is returned to the fieldwork recruiter upon completion

The fieldwork recruiter checks the short screening questionnaire

Fulfil the sensitive inclusion criteria

Does not fulfil the sensitive inclusion criteria

Thank the man for his time and approach another man

Show and explain the participant information sheet to the man

Then obtain oral consent from the man to participate in the study

Agree to participate in the study

Refuse to participate in the study

Pass the self-administered questionnaire to the man

Thank the man for his time and approach another man
