# Supplementary material for: What are the factors associated with human immunodeficiency virus/sexually transmitted infection screening behaviour among heterosexual men patronising entertainment establishments who engaged in casual or paid sex? – Results from a cross-sectional survey in an Asian urban setting
Source: BMC Infect Dis. 2016 Dec 19;16:763. doi: 10.1186/s12879-016-2088-8 (PMC5168707; doi:10.1186/s12879-016-2088-8)
Supplement: Additional file 2: — Appendix II. Outcome, sociodemographic factors, HIV knowledge and sexual behaviour amongst heterosexual men by geographical site. (DOC 114 kb) [file 12879_2016_2088_MOESM2_ESM.doc]

| **Factor** | **Clarke Quay**  **(n=302)** | **Tanjong Pagar**  **(n=302)** | **P value** |
| --- | --- | --- | --- |
| **Outcome** |  |  |  |
| Gone for HIV or STI testing in the past 6 months |  |  |  |
| No | 215 (71.2) | 226 (74.8) | 0.31 |
| Yes | 87 (28.8) | 76 (25.2) |  |
|  |  |  |  |
| **Sociodemographics** |  |  |  |
| Ethnicity |  |  |  |
| Chinese | 185 (61.3) | 192 (63.6) | 0.56 |
| Non-Chinese | 117 (38.7) | 110 (36.4) |  |
|  |  |  |  |
| Marital status |  |  |  |
| Single | 271 (89.7) | 221 (73.2) | <0.001 |
| Married | 31 (10.3) | 81 (26.8) |  |
|  |  |  |  |
| Highest education level* |  |  |  |
| No formal education/primary/secondary | 30 (10.0) | 60 (19.9) | <0.001 |
| Institute of technical education/A Level/ Diploma | 186 (62.0) | 146 (48.3) |  |
| University/Post-graduate | 84 (28.0) | 96 (31.8) |  |
|  |  |  |  |
| Housing type |  |  |  |
| 1-3 room public housing | 44 (14.6) | 47 (15.6) | 0.91 |
| 4-5 room public housing | 175 (57.9) | 170 (56.3) |  |
| Private property | 83 (27.5) | 85 (28.1) |  |
|  |  |  |  |
| Occupation |  |  |  |
| Not currently employed | 63 (20.9) | 39 (12.9) | 0.02 |
| Blue-collar job | 70 (23.2) | 87 (28.8) |  |
| White-collar job | 169 (55.9) | 176 (58.3) |  |
|  |  |  |  |
| Age in years, median (IQR) | 25 (22 – 29) | 28 (25 – 35) | <0.001 |
|  |  |  |  |
| **HIV knowledge** |  |  |  |
| HIV-KQ-18 Knowledge Score, median (IQR) | 14 (11 – 15) | 13 (10 – 15) | 0.24 |
|  |  |  |  |
| **Sexual behaviour** |  |  |  |
| Number of partners in the past 6 months, median (IQR) | 4 (2 – 5) | 4 (2 – 6) | 0.02 |
|  |  |  |  |
| Engaged in anal sex with casual or paid partner in the past 6 months | |  |  |
| No | 252 (83.4) | 248 (82.1) | 0.67 |
| Yes | 50 (16.6) | 54 (17.9) |  |
|  |  |  |  |
| Type of casual/paid partners in the past 6 months |  |  |  |
| More than 1 type of casual/paid partner | 76 (25.2) | 80 (26.5) | 0.02 |
| Sex workers from brothels only | 14 (4.6) | 13 (4.3) |  |
| Casual partners only | 197 (65.2) | 173 (57.3) |  |
| Female entertainment workers only | 15 (5.0) | 36 (11.9) |  |
|  |  |  |  |
| Type of regular partners in the past 6 months |  |  |  |
| Without any regular partner | 184 (60.9) | 146 (48.3) | <0.001 |
| With wife as regular partner only | 13 (4.3) | 40 (13.3) |  |
| With girlfriend/mistress as regular partner only | 98 (32.5) | 100 (33.1) |  |
| With both wife and girlfriend/mistress as regular partners | 7 (2.3) | 16 (5.3) |  |
|  |  |  |  |
| Partner asking to use a condom all the time in the past 6 months | | | |
| No | 157 (52.0) | 129 (42.7) | 0.02 |
| Yes | 145 (48.0) | 173 (57.3) |  |
|  |  |  |  |
| Consistent condom use with casual or paid partner in the past 6 months | | | |
| No | 164 (54.3) | 156 (51.7) | 0.51 |
| Yes  **Additional file 2: Appendix II. Outcome, sociodemographic factors, HIV knowledge and sexual behaviour amongst heterosexual men by geographical site** | 138 (45.7) | 146 (48.3) |  |
|  |  |  |  |
| Condom use at last sex with a casual or paid partner | | | |
| No | 125 (41.4) | 115 (38.1) | 0.41 |
| Yes | 177 (58.6) | 187 (61.9) |  |

All figures in the table refer to frequency (column percentage) unless otherwise indicated

*Contains missing value of 2 for highest education level
